# The preferences of breast cancer patients regarding a digital social care platform

**DOI:** 10.1186/s12905-025-03792-2

**Published:** 2025-05-21

**Authors:** Sima Rafiei, Peigham Heidarpoor, Saber Souri, Zahra Nejatifar, Mohammad Amerzadeh

**Affiliations:** 1 Social Determinants of Health Research Center, Research Institute for Prevention of Non- Communicable Diseases, Qazvin University of Medical Sciences, Qazvin, Iran; 2 Department of Community Based Education of Health Sciences, School of Medical Education and Learning Technologies, Shahid Beheshti University of Medical Sciences, Tehran, Iran; 3 Student Research Committee, School of Public Health, Qazvin University of Medical Sciences, Qazvin, Iran

**Keywords:** Digital health, Breast cancer, Social care, Patient preferences, Discrete choice experiment, Conjoint analysis

## Abstract

**Background:**

Breast cancer is a multifaceted condition affecting women globally, with patients often facing emotional, psychological, and social challenges. This study explored breast cancer patients’ preferences for features of a digital social care platform.

**Methods:**

A cross-sectional study using Conjoint Analysis (CA), grounded in economic utility theory, was conducted. A Discrete Choice Experiment (DCE) questionnaire was administered to 158 breast cancer patients at a university hospital in Iran between November 2023 and January 2024. Data were analysed using ordered logistic regression in Stata 13.

**Results:**

All platform attributes significantly influenced preferences (*P* ≤ 0.05). Emotional support had the highest impact (β = 1.132), followed by informational (β = 0.973) and esteem support (β = 0.864). Instructional videos increased the likelihood of choosing a digital platform 2.45 times compared to text-based messages (*P* < 0.001). Personalized online support was 1.42 times more preferred than generic supportive messages (*P* < 0.001). Mindfulness affirmations were 1.14 times more preferred than inspirational messages (*P* < 0.001).

**Conclusion:**

Digital tools that prioritize emotional, informational, and esteem support—especially through personalized online support and instructional videos can improve patient engagement and acceptability. These findings support the patient-centred design of digital social care platforms to enhance quality of life for breast cancer patients.

**Supplementary Information:**

The online version contains supplementary material available at 10.1186/s12905-025-03792-2.

## Background

Breast cancer is a prevalent and complex condition affecting millions of women worldwide, not only posing physical challenges but also having significant emotional, psychological, and social implications. A diagnosis often disrupts a patient’s personal, familial, and occupational life, underscoring the need for holistic care that extends beyond clinical treatment [1, 2].

Studies have shown that individuals with breast cancer are at increased risk of anxiety, depression, and social isolation [3, 4]. They experience a higher prevalence of psychological problems compared to the general population [5]. Research emphasises that the journey of breast cancer involves more than medical treatment, requiring a comprehensive approach that addresses psychosocial needs as well [6].

Social support plays a pivotal role in mitigating psychological burden, improving emotional resilience, and enhancing overall well-being [7]. Support may come in various forms of emotional, informational, instrumental, and appraisal, and can be offered by healthcare professionals, family members, peers, or digital sources [8, 9].

However, access to consistent and personalized support is often limited, particularly for women in underserved or remote regions. Traditional support mechanisms such as in-person counselling, support groups, or family caregiving may be constrained by geographic, logistical, or socio-economic barriers. Given the proven benefits of social support in managing breast cancer, digital platforms present a promising solution to extend these benefits in scalable, accessible, and cost-effective ways [10].

Given the proven benefits of social support, digital platforms offer a scalable solution to extend these benefits remotely and continuously, overcoming many logistical challenges [11]. Recent years have seen a significant rise in digital interventions aimed at supporting cancer patients, especially during the COVID-19 pandemic when traditional healthcare access was disrupted [12, 13].

Despite growing interest in digital support tools, few studies have investigated which platform features are most valued by breast cancer patients, particularly in low- and middle-income countries (LMICs), where resource constraints further limit access to in-person care [14]. Understanding user preferences is crucial for designing effective and engaging platforms.

This study addresses this gap by exploring the preferences of Iranian breast cancer patients for different attributes of a digital social care platform. Using Conjoint Analysis (CA) and a Discrete Choice Experiment (DCE) grounded in economic utility theory, we aim to identify the support features that patients value most, providing evidence to guide the patient-centred design of future digital interventions.

### Theoretical framework

To guide the development of our digital social care platform, we adopted the Persuasive Systems Design (PSD) model. This framework, grounded in behavioural science and communication theory, provides a structured approach for designing technologies that influence user attitudes and behaviours in ethical and evidence-based ways [15]. The PSD model categorizes 28 persuasive design principles into four domains: primary task support, dialogue support, system credibility support, and social support. In this study, these categories were used to guide the development of platform features aligned with the psychological and emotional needs of breast cancer patients [16].

The primary task support category involves features that help users effectively engage with the core functionality of the system, encouraging routine usage and supporting self-management. In our platform, this was reflected through regular delivery of practical, context-specific tips—such as coping strategies for treatment side effects and user-friendly tracking tools that allow patients to monitor their own emotional wellbeing and social engagement. By facilitating ongoing and personalized interaction, the design promotes a sense of consistency and sustained support [16].

Dialogue support principles focus on system interactivity and timely feedback to reinforce motivation and behaviour change. In this category, we incorporated esteem-enhancing features, such as motivational messages tailored to individual progress and interactive check-ins designed to prompt emotional reflection. These were complemented by mindfulness prompts and automated affirmations, offering psychological reinforcement to maintain engagement and alleviate distress [16, 17].

In the domain of system credibility support, we emphasized the importance of building trust and ensuring the perceived reliability of the platform. This was achieved by integrating instructional videos based on verified medical sources and clearly indicating the credentials of content contributors, such as oncologists and licensed psychologists. Additionally, supportive educational content was sourced or adapted from reputable organizations like the World Health Organization, further reinforcing the platform’s authority [17].

Finally, the social support category drew upon principles that leverage social influence and community dynamics to foster user motivation. Here, we embedded features such as moderated discussion forums where users could share experiences and advice, peer-matching systems that encouraged regular check-ins between participants, and interactive challenges aimed at promoting a sense of community and shared resilience. These features were specifically designed to reduce feelings of isolation and strengthen patients’ sense of belonging [15–17].

By operationalizing PSD principles into concrete, user-informed features, we aimed to develop a persuasive, trustworthy, and emotionally supportive digital environment. This approach not only aligns with the theoretical foundations of persuasive technology but also reflects a commitment to patient-centred design in digital health interventions.

### Review of the literature

Digital health encompasses a range of information and communication technologies designed to improve health outcomes efficiently and cost-effectively [18]. Research supports that mobile applications can assist cancer patients in self-management by providing timely access to credible information and facilitating navigation through treatment pathways.

For instance, Buscemi et al. conducted a randomized controlled trial with 50 breast cancer survivors using a mobile app called My Guide, which was designed to provide culturally tailored psychosocial support. They found significant improvements in breast cancer knowledge and patient satisfaction [19]. Similarly, in a cohort study involving 4,475 breast cancer patients, mobile app usage was associated with increased medication adherence, suggesting digital tools can improve treatment compliance at scale [20].

Ponder et al. assessed the psychological outcomes of a mobile app used perioperatively by breast cancer patients (*n* = 57), reporting significant reductions in anxiety and depression after app use, which they attributed to improved informational and emotional support [21]. Wyatt et al. surveyed 20 breast cancer patients using a web-based symptom monitoring platform, noting that enhanced communication and symptom awareness increased their confidence in decision-making and adherence to treatment [22]. In Taiwan, a longitudinal study involving 98 cancer patients showed that using a self-management mHealth app significantly improved quality of life over three months [23].

While these studies confirm the general benefits of digital health interventions, many focus on clinical symptom management or informational tools. There is limited attention to platforms offering social care support, defined as emotional reassurance, esteem-building, and peer connection tailored to the unique psychosocial needs of breast cancer patients. This distinction is crucial because social care features, such as peer interaction, community belonging, and emotional validation, directly influence user engagement and mental well-being, especially in chronic illness contexts [24]. The sustained use of digital platforms depends not only on medical relevance but also on meaningful social support delivered through persuasive design features.

Persuasive System Design (PSD) offers a promising framework for developing these digital interventions. PSD categorizes persuasive features into primary task support, dialogue support, system credibility, and social support, aligning well with the behavioural and emotional needs of cancer patients [16, 25]. However, a critical gap remains it is still unclear how to translate PSD principles into features that patients truly value. For instance, do patients prefer automated reminders, or would moderated peer communities be more effective in maintaining engagement. These practical questions remain underexplored, limiting the scalability and personalization of digital social care platforms.

However, literature supports the idea that incorporating local values, needs, and expectations improves digital health intervention adoption [2]. Engaging users early in the design process is essential to ensure relevance and personalization. Therefore, exploring breast cancer patients’ preferences for social platform features is vital to operationalize PSD in ways that truly resonate.

This research addresses this gap in preference-based design, aiming to develop a deeper understanding of the types of support features—emotional, esteem, informational—that patients prioritize in a digital environment. Ultimately, this knowledge will contribute to building patient-centred, persuasive social care platforms that enhance both engagement and psychosocial outcomes.

## Methods

### Study design

This cross-sectional study used Conjoint Analysis (CA), grounded in economic utility theory, to examine breast cancer patients’ preferences for features of a digital social care platform. Preferences were elicited using a Discrete Choice Experiment (DCE), which allows for the estimation of the relative importance of various service attributes [26].

### Study participants and the sampling method

A sample of 158 breast cancer patients was recruited from the outpatient oncology unit of a tertiary hospital affiliated with Qazvin University of Medical Sciences in Iran. Recruitment occurred over a three-month period (November 2023 to January 2024), using consecutive sampling.

Sample size was determined using Johnson and Orme’s widely accepted rule of thumb for conjoint experiments (nta/c ≥ 500), where n = number of respondents, t = number of tasks, a = number of alternatives per task, and c = the maximum number of levels for any attribute [27]. This yielded a minimum requirement of 62 participants. Accounting for a potential response rate of 42%—as reported in Merlo et al.—we recruited 158 participants to ensure adequate statistical power.

Inclusion criteria were: diagnosis of Stage I or II breast cancer within the past two years, currently undergoing chemotherapy and/or radiotherapy, no history of other chronic physical or psychological disorders, and willingness to provide informed consent. Patients who declined participation or were deemed emotionally distressed were excluded.

All participants (or their legal guardians) received an information sheet detailing the study purpose, procedures, confidentiality, and their right to withdraw at any time. Informed written consent was obtained.

### Translation of PSD principles into the features of a digital social support system

Persuasive System Design (PSD) principles were translated into functional features aligned with five domains of social support as proposed by Cutrona and Suhr: informational, emotional, esteem, tangible, and social network support. The mapping of PSD categories into platform features is shown in Table 1. For instance, esteem support was operationalized via inspirational messages and mindfulness prompts, while tangible support was represented by options such as automated medication reminders and real-world service assistance for bedridden patients.

### Conjoint design

To identify the key attributes that make breast cancer patients opt into a digital social care system based on a persuasive system design model and social care system, we used the information from the literature review to find a suitable model for designing a persuasive technology. We further examined which features had the most significant role in stimulating motivation and adherence to the digital system among patients with breast cancer. The literature review led to a broad range of potential attributes, narrowed to five based on a social support category system developed by Cutrona and Suhr [28]. Afterward, for each attribute, the research team selected realistic, relevant, and easy-to-understand levels through which patients were asked to choose from. Therefore, eight experts discussed a preliminary list of attributes, including social medicine specialists, health services management, and health policy-making experts. The experts were selected using the purposive sampling technique, and the panel discussion was conducted at Qazvin University of Medical Sciences in October 2023. The panel discussed the features of a digital social support system and tried to suit them according to the four main categories of PSD principles. Consensus was reached by the experts during the first panel, resulting in the selection of five attributes and ten levels [29]. Table 1 displays the attributes and their levels established in this study.

**Table 1:** Attributes and associated levels

PSD Principles	Category	Sub-category	Attribute	Levels
Primary task support	Emotional support	-Relationship -Sympathy -Encouragement -Empathy	Provision of social support and caring messages	-Send regular sympathy and caring messages by automated SMS text or MMS messages -Provide online personal support workers to checkup
	Tangible support	-Physically available goods and/ or services	Provision of direct material aid or other concrete assistance	-The ability of the application to send someone to help a patient if he/she is confined to bed - Send medication reminder
Social support	Social network support	Companionship^1^	Provision of messages that strengthen individual’s sense of belonging to peers or people with similar interests or situations	-Provide contact details of available social care services -Offer chat groups with peers
Dialogue support	Esteem support	-Compliment^2^ -Feedback	Provision of esteem support messages aiming to increase an individual’s skills, abilities, and confidence	-Send inspirational messages to patients by automated SMS text or MMS messages *-* Offer mindfulness and positive affirmations
System credibility	Information support	-Teaching -Reference	Provision of informative support^3^	- Use SMS for sending truthful informative quotes or messages - Use instructional web-based videos

^1^ Gathering people with the same experience and/ or situation together

^2^ Using compliments, validation, and positive feedback with users to increase persuasion

^3^ Providing truthful and reliable information

#### Scenario development

In the scenario development phase, determined attributes and levels would lead to a manageable number of scenarios based on the fractional factorial design. In this study, all five attributes had two levels. Thus, the total number of scenarios for five attributes was estimated to be 2^5^ = 32 in a complete factorial design. As not all possible scenarios can be included in the CA, the fractional factorial design was adopted using the SPSS software version 20 to obtain a manageable level while capturing essential information about the system’s behavior. Using this design, the possible scenarios were reduced to 16.

#### Administering the questionnaire, and data collection

The questionnaire comprised three main sections. The first section presented an introduction and explanation of the attributes and their levels to help respondents better understand them. In the second section, patients were presented with 16 scenarios and were asked to rate them from 1 to 10, with 1 representing low desirability and 10 representing high desirability. The last section was about the socio-demographic details of respondents, including their age, marital status, family size, educational level, and occupation. (Appendix)

#### The validity and reliability of the questionnaire

Before completing the questionnaire, a trained researcher explained the choice tasks to study participants to ensure their comprehension of information when making choices. This explanation and face-to-face interviews based on the think-aloud methods were used to confirm that the scenarios in a DCE were understandable for participants before deploying the final survey [30]. The primary purpose of interviews was to examine the face validity of the questionnaire and assess the understanding of the wording used to characterize the study attributes and levels. To apply a think-aloud approach, a pilot of 20 patients was asked to complete the choice tasks before implementing the survey. In case of any ambiguity, while answering the questionnaire, the researcher tried to document all raised issues and discuss specific aspects of the survey to identify any modification and necessary changes in the wording or displaying of the survey questions [31]. To check reliability, we used a pretesting approach with a pilot of 20 patients to understand whether the choice experiment was performing as intended and ensure the relevance and comprehensiveness of the DCE [32]. Accordingly, we checked two important items: [1] whether the attributes represent the concept of interest; and [2] whether attribute levels are appropriate and arranged logically. Regarding the comprehension of the DCE, we also checked whether key terms are consistently comprehensible and whether participants can visualize proposed scenarios in a related context in which they are asked to make choices. Finally, in the elicitation of the DCE, we checked how the process of creating a choice is being undertaken by study participants, whether underlying mental shortcuts contribute to decision-making, and whether participant preferences reflect their choices properly [33].

#### Data analysis

Data were analyzed using STATA version 13. Ordered Logistic Regression was applied to assess the effect of attribute levels on patients’ scenario ratings. The ordinal dependent variable ranged from 1 to 10, representing levels of desirability. Independent variables included scenario attribute levels and patient demographic characteristics.

To ensure validity of model assumptions, residual distributions were inspected, and the proportional odds assumption was verified using the Brant test. Normality of residuals was deemed satisfactory for the application of the ordered logistic regression model with maximum likelihood estimation. Significance was determined at a threshold of *p* ≤ 0.05.

Assuming that the errors (residuals) of the model are approximately normally distributed, ordered logistic regression with maximum likelihood estimation was the right analytical approach for the analysis (Eq. 1) [34]. The starting point for the ordered logistic regression model is Eq. 1.

$${V_i}\left( {{P_h}} \right) = {b_1}{Z_{1h}} + {b_2}{Z_{2h}} + \ldots + {b_7}{Z_{7h}} + {c_{1h}}{X_1} + \ldots + {c_{Rh}}{X_R} + {\varepsilon _{ih}}$$

In the above equation, *Ph* is the h-th scenario; *Vi*(*Ph*)is the measure of utility that a patient derives from the attributes of the h-th scenario; *Z*1*h*… *Z*7*h* is a vector for the levels of observed attributes; *X*1… *XR* is a vector of the patient’s characteristics; *b*1… *b*7 and *c*1*h*… *c*Rh are unknown parameters, and *ε*ih is a random error. The ratings between 1 and 10 were observable, where:

Rating = 1 if *P*_*h*_ ≤ µ1.

Rating = 2 if µ1 < *P*_*h*_ < µ2.

Rating = 3 if µ2 < *P*_*h*_ < µ3.

Rating = 4 if µ3 < *P*_*h*_ < µ4.

Rating = 5 if µ4 < *P*_*h*_ < µ5.

Rating = 6 if µ5 < *P*_*h*_ < µ6.

Rating = 7 if µ6 < *P*_*h*_ < µ7.

Rating = 8 if µ7 < *P*_*h*_ < µ8.

Rating = 9 if µ8 < *P*_*h*_ < µ9.

Rating = 10 if *P*_*h*_ < µ9 (µ1… µ9 are estimated cutoff points).

## Results

Results depicted that the mean age of study participants was 46 (SD = 6.4). Most were married (69.7%), and their family size was between 2 and 4 people. Regarding employment, the majority were unemployed (68.99%) and had diplomas (72.1%) (Table 2).

**Table 2:** Study participants’ characteristics

Characteristics		Frequency (%)
Marital status	Single	48(30.3)
	Married	110(69.7)
Family size	≤ 2	17(10.7)
	2–4	88(55.6)
	≥ 5	53(33.7)
Educational level	Under diploma	26(16.4)
	Diploma	114(72.1)
	University degree	18(11.5)
Employment	Unemployed	109(68.99)
	Employed	49(31.01)
Treatment type	Chemotherapy	77(48.7)
	Radiotherapy	12(7.5)
	Chemotherapy and radiotherapy	69(43.8)
		**Mean (SD)**
Age		46(6.4)

Study findings also revealed that all attribute levels significantly affected patients’ preferences (*P* ≤ 0.05). The estimated coefficients show that “emotional support” had the most significant effect on patients’ preferences (β = 1.132), followed by “information support” (β = 0.973) and “esteem support” (β = 0.864). (Table 3).

**Table 3:** Regression modelling of hypothetical scenarios

Attribute levels		Unstandardized Coefficients	Standard Error	Z	Odds Ratio	*P* value
Information support	Informative quotes or messages (baseline)	0.973	0.055	17.61	2.45	0.001
	Instructional videos					
Emotional support	Sympathy and caring messages (baseline)	1.132	0.052	16.72	1.42	0.001
	Online personal support					
Esteem support	Inspirational messages (baseline)	0.864	0.057	13.52	1.14	0.021
	Mindfulness affirmation					
Social network support	Contact details (baseline)	0.677	0.055	10.74	1.62	0.033
	Peer chat					
Tangible support	Physical help (baseline)	0.822	0.049	12.88	0.98	0.075
	Medication reminder					

The odds ratios shown in Table 3 depict that the “provision of instructional videos” would increase the likelihood of accepting and using the digital social care application among study participants almost 2.5 times more compared to the “provision of informative quotes or messages” (*P* < 0.05). Furthermore, a scenario that allowed patients to get access to “online personal support” was 1.42 times more likely to be preferred by patients than “providing them sympathy and caring messages” (*P* < 0.05). Regarding esteem support strategies, a scenario that provided patients with “mindfulness affirmations” was 1.14 times more likely to be preferred than a scenario that provided them with “inspirational messages” (*P* < 0.05).

Marginal effects estimate the discrete change in the probability of choosing a digital support application when the attribute level changes. For example, providing instructional videos increased the probability of a scenario being chosen by 17%, reflecting a strong patient preference for visual learning content. Access to online personal support was associated with a 12% increase, while mindfulness affirmations led to an 18% increase in preference (Table 4).

**Table 4:** Take-up rates for digital social care application under different options

Attributes	Levels	Marginal effects	Take-up rates	*P* value
Information support	Informative quotes or messages	0.17	1	< 0.001
	Instructional videos		17	
Emotional support	Sympathy and caring messages	0.12	1	< 0.001
	Online personal support		12	
Esteem support	Inspirational messages	0.18	1	0.005
	Mindfulness affirmation		18	
Social network support	Contact details	0.08	1	0.045
	Peer chat		8	
Tangible support	Physical help	0.26	26	0.2
	Medication reminder		1	

## Discussion

The study aimed to explore the preferences of breast cancer patients for the features that constitute a preferable digital social care platform. Study findings indicate that emotional support significantly impacts patients’ preferences, followed by information support and esteem support. These findings are consistent with a similar study that reported that social support, including emotional support from family members, positively affected breast cancer patients’ psychological well-being and social functioning [34]. Solid social support, such as family bonds and quality relationships, enhances the mental adjustment of patients. Additionally, factors such as employment and higher social status of individuals revealed a significant role in enabling them to better adapt to changes in their physical, psychological, and emotional conditions [35]. Likewise, another study reported that increased social support, particularly regarding network size and social connection, was associated with better survival rates among patients with breast carcinoma [36]. Furthermore, breast cancer patients who perceived moderate-to-high levels of social support experienced reduced severity of chemotherapy-related symptoms compared to those with low perceived social support. Expressing emotional support on social media platforms has also enhanced cancer patients’ coping strategies by increasing their perception of bonding [37]. Close relationships, such as marital confiding and dependable nonhouseholder support, as well as emotional processing, have been connected to decreased mortality rates in women with breast cancer. Moreover, the provision of emotional support and implementation of organizational changes in nursing care facilities could improve breast cancer patient’s sense of safety and security, enabling them to plan for the future despite their challenging circumstances [38]. These findings emphasize the significant role of social support, particularly emotional support, in improving the well-being, coping strategies, and treatment outcomes among breast cancer patients. Factors such as family support, social interactions, employment, and social status of patients can motivate patients to restore balance in life and evolve positively. Our study findings also indicated that providing social care support for breast cancer patients through digital apps could significantly increase the likelihood of their engagement with and acceptability of these technologies. These findings align with the literature demonstrating that pre-visit microlearning breast cancer educational videos significantly impacted patient satisfaction and engagement with the technology [36]. In a randomized controlled trial, video preparation for breast cancer treatment may support reduced distress and increased preparedness, particularly among vulnerable or underserved patients [36]. Additionally, incorporating some features, including background music, an easy-to-understand language, and short-term health videos promoting breast cancer education, improved the knowledge absorption efficiency significantly [17]. Likewise, personalized video education before the first oncologic consultation was feasible and beneficial for breast cancer patients, particularly in explaining complex contents like cancer subtypes and their treatment procedures [39]. Another study highlighted that interactive technologies and videotapes for cancer education could increase patients’ knowledge and awareness of the disease. Sustainable communication with health professionals brought them higher satisfaction levels [40]. Similarly, women with breast cancer expressed that computer-mediated support groups provided unique support, facilitating communication and offering emotional, encouraging, and informational support [41]. Moreover, breast cancer patients engaging in social support exchanges on Facebook formed a peer community that helped buffer stress effects from negative health experiences during diagnosis and transitioning off cancer therapy. These findings emphasize the effectiveness of instructional videos in increasing the acceptance and utilization of digital social care applications among breast cancer patients. Using apps as supportive tools has demonstrated positive outcomes, including improved patient satisfaction, engagement, and preparedness [42]. Incorporating interactive elements and personalization further enhances the educational experience. Additionally, digital platforms, such as computer-mediated support groups and social media communities, serve as valuable sources of support, fostering communication and providing emotional and informational assistance for women with breast cancer [43].

Our results indicate that patients with access to instructional videos instead of merely receiving regular informative messages had a 17% higher probability of choosing digital social care support. Additionally, our study findings confirmed that the opportunity to receive personal online support increased the likelihood even further by 12%. A similar study revealed that perceived social support has a positive impact on the quality of life for younger breast cancer survivors. In contrast, uncertainty has a negative effect, accounting for 27.20% of the variance in life quality [44]. Moreover, participation in a Twitter breast cancer support group increased patients’ perceived knowledge and decreased anxiety. However, it is essential to consider technology access and health literacy issues when developing online digital videos for cancer prevention and education [45]. Viewing a videotape of breast cancer educational materials can potentially influence patients’ preferences toward breast-sparing surgery, while both booklets and videos can effectively increase their knowledge about treatment options. Furthermore, digital support groups for breast carcinoma showed promise in improving individuals’ quality of life, reducing psychological symptoms, and enhancing coping skills [46]. Internet-based breast cancer support groups also played a crucial role in eliminating social isolation and empowering women by facilitating knowledge exchange and sharing experiences. Literature supports that digital platforms can provide health messages, reminders, essential support, or behaviour monitoring. The remaining topics include particular support to patients with unique health goals, such as a healthy diet, physical activity, smoking cessation, disease prevention, or engagement in behaviour monitoring to track health behaviours and outcomes [47]. Perceived social support is crucial for the well-being of breast cancer survivors, and participation in online support groups can offer knowledge, reduce anxiety, and enhance coping. However, the efficacy of online support groups may vary, and considerations such as technology access, health literacy, and distress levels should be considered when designing and implementing these interventions [46].

The non-significant *P*-value for “Tangible Support” indicates that the difference between providing physical help and medication reminders did not significantly influence patients’ preferences for the digital social care platform. This result suggests that patients may view both options as equally important, or that their preferences for tangible support may be context-dependent and vary based on individual circumstances. This may reflect a preference for emotional over practical aid in a digital context, warranting further study into the role of tangible support in digital platforms for breast cancer patients [48].

The findings of a review acknowledged that the acceptability of these platforms is an important consideration for developers and policymakers, which necessitates the application of a suitable method to explore users’ preferences for the features of a digital social care platform. This study applied CA to evaluate patients’ preferences for different platform attributes without directly asking them to state their preferred options. This method has been used in different areas such as priority setting, determining desirable patient treatments, evaluating different healthcare alternatives, and exploring patients’ preferences in the physician-care recipient relationship [26]. Such a method allows the ranking of health services when setting priorities and estimating the relative importance of different service features. Relative results enable policymakers to evaluate each feature’s impact on a particular strategy’s overall advantage [49].

The findings of this research provide valuable practical insights to guide the design and real-world implementation of effective digital social care platforms tailored to the preferences of breast cancer patients. By prioritizing the most preferred features identified, such as emotional support, informational support with instructional videos, esteem support through mindfulness exercises and positive affirmations, and personalized online support from healthcare professionals or peers, developers can create platforms that resonate with patients and increase their engagement. Incorporating these elements as core components can foster a sense of community, alleviate isolation, enhance disease self-management, and promote resilience throughout the cancer journey. However, successful implementation requires a multi-faceted approach [50]. Healthcare organizations should actively involve breast cancer patients and survivors through focus groups, usability testing, and iterative feedback loops to continuously refine and co-design platforms that evolve to meet changing needs over time. For example, focus groups could identify preferred video content, while usability testing ensures accessibility for diverse patient populations. Comprehensive training programs for staff and patients, integration into existing care pathways, robust data privacy and security protocols, and collaboration with patient advocacy groups, community organizations, and technology partners are crucial for effective dissemination and adoption [51].

### Study limitations

This study had several limitations that need to be acknowledged. First, the sample was drawn from a single hospital in Iran, which might restrict the generalizability of study findings to other geographic regions and healthcare settings. Future multi-center studies with larger and more diverse patient populations are suggested. Moreover, although CA is a robust method for eliciting preferences, the hypothetical nature of the choice scenarios might not accurately reflect real-world decision-making. Accordingly, longitudinal studies examining patients’ uptake and engagement with digital social care platforms based on the preferred features would be valuable. Additionally, this study focused specifically on breast cancer patients. Since the need for social support might vary across different cancer types and stages, it necessitates future research studies to explore the preferences of patients with other cancer diagnoses. Understanding the unique needs and priorities of various patient groups will inform the development of tailored digital interventions.

## Conclusion

In conclusion, our study highlights the potential effectiveness of digital social care apps and personalized online support in enhancing patient engagement and acceptability. Specifically, providing instructional videos, rather than regular informative messages, significantly increased the likelihood of patients choosing digital social care support. Additionally, offering personalized online support further enhanced this likelihood. These findings suggest that incorporating digital tools and tailored support within healthcare platforms could help increase patient engagement, potentially enhancing their well-being during the disease journey. By understanding which features encourage patients to accept and engage with digital platforms, developers can better tailor these tools to meet patient preferences and improve practical usage.

While our study focuses on patient preferences, it is possible that features such as instructional videos and personalized support may contribute to reducing feelings of loneliness and stress by fostering greater engagement. However, further research is needed to confirm these outcomes in real-world settings.

The implications of this study are significant for healthcare policymakers, practitioners, and technology developers. As digital health solutions continue to evolve, designing platforms that align with patient needs and preferences is crucial. Policymakers should prioritize integrating patient-centered design principles and allocate resources to developing tailored digital social care solutions. Healthcare practitioners can leverage these insights to better understand the types of support that resonate with patients, thereby improving communication, shared decision-making, and overall engagement.

For technology developers, this study underscores the importance of co-designing digital health platforms with end-users. By actively involving patients in the design process and iterating based on their feedback, platforms are more likely to gain adoption and sustained use. Collaborative efforts between healthcare organizations, patient advocates, and technology companies will be vital to creating user-friendly, evidence-based, and effective digital social care solutions.

Future research should validate these preferences and explore their real-world application to ensure the development of digital tools that effectively support patients throughout their cancer journey.

## Electronic supplementary material

Below is the link to the electronic supplementary material.

Supplementary Material 1

## Data Availability

The datasets used and/or analyzed during the current study available from the corresponding author on reasonable request. The entire dataset is in Farsi language. The Data can be available in English language for the readers and make available from the corresponding author on reasonable request.
