# Supplementary material for: The preferences of breast cancer patients regarding a digital social care platform
Source: BMC Womens Health. 2025 May 21;25:240. doi: 10.1186/s12905-025-03792-2 (PMC12093778; doi:10.1186/s12905-025-03792-2)
Supplement: Supplementary file 1 — Supplementary Material 1 [file 12905_2025_3792_MOESM1_ESM.pdf]

Dear respondent

We believe that breast cancer is a multifaceted condition that encounters patients with various physical, emotional, psychological, and social issues throughout their life. Our hypothesis is that an appropriate digital social support system for breast cancer patients can effectively help them cope with the disease and its treatment process. Thus, we designed this study to elicit the preferences of you as potential users of the platform for the features that constitute a preferable digital social care system. We really appreciate your contribution in this study and hope your truthful information helps us develop a useful digital platform which enables the provision of social support for cancer patients including informational, emotional, esteem, social network support, and tangible support.

After reading the explanation, please read the 16 scenarios and rate them from 1 to 16, with 1 representing low desirability and 16 representing high desirability. The last section was about the socio-demographic details of you, including their age, marital status, family size, educational level, and occupation. That would be great if you also answer this part properly.

Before going through the 16 scenarios, please read a short explanation given below to gain more insight about different features of a digital system for social support services. The support system involves five main domains of social support: (1) informational, (2) emotional, (3) esteem, (4) social network support, and (5) tangible support. Informational support refers to providing informative messages that contain knowledge, advice, or feedback; it also might include some instructional videos to give necessary information to patients about their disease, and treatment journey. Emotional support is about providing sympathy, empathy, and caring messages or providing online mood checkup. Esteem support refers to sending inspirational messages or offering mindfulness and positive affirmations aiming to increase an individual's skills, abilities, and confidence. Social network support is about the activities that strengthen an individual's sense of belonging to peers or people with similar interests or situations. This might be achieved through the provision of contact details of healthcare professionals, or peer group chat rooms to facilitate sharing lived experiences. Finally, tangible support is considered to provide required goods and services to patients. This is called as "direct material aid" in our questionnaire with two different features: first, sending someone to help the patient with daily life activities, and house chores; second, sending medication reminder.

Option 1
**Information support:** Sending informative messages
**Social support:** Sending automated caring messages
**Esteem support:** Sending automated inspirational messages
**Sense of belonging:** Provision of contact details
**Material aid:** medication reminder

Option 2
**Information support:** Sending instructional videos
**Social support:** Sending automated caring messages
**Esteem support:** Sending automated inspirational messages
**Sense of belonging:** Provision of contact details
**Material aid:** Send someone to help

***

Option 3
**Information support:** Sending informative messages
**Social support:** Online mood checkup
**Esteem support:** Sending automated inspirational messages
**Sense of belonging:** Provision of contact details
**Material aid:** Send someone to help

Option 4
**Information support:** Sending instructional videos
**Social support:** Online mood checkup
**Esteem support:** Sending automated inspirational messages
**Sense of belonging:** Provision of contact details
**Material aid:** medication reminder

***

Option 5
**Information support:** Sending informative messages
**Social support:** Sending automated caring messages
**Esteem support:** Offer mindfulness and positive affirmations
**Sense of belonging:** Provision of contact details
**Material aid:** Send someone to help

Option 6
**Information support:** Sending instructional videos
**Social support:** Sending automated caring messages
**Esteem support:** Offer mindfulness and positive affirmations
**Sense of belonging:** Provision of contact details
**Material aid:** medication reminder

***

Option 7
**Information support:** Sending informative messages
**Social support:** Online mood checkup
**Esteem support:** Offer mindfulness and positive affirmations
**Sense of belonging:** Provision of contact details
**Material aid:** medication reminder

Option 8
**Information support:** Sending instructional videos
**Social support:** Online mood checkup
**Esteem support:** Offer mindfulness and positive affirmations
**Sense of belonging:** Provision of contact details
**Material aid:** Send someone to help

***

Option 9
**Information support:** Sending informative messages
**Social support:** Sending automated caring messages
**Esteem support:** Sending automated inspirational messages
**Sense of belonging:** Chat groups
**Material aid:** Send someone to help

Option 10
**Information support:** Sending instructional videos
**Social support:** Sending automated caring messages
**Esteem support:** Sending automated inspirational messages
**Sense of belonging:** Chat groups
**Material aid:** medication reminder

***

Option 11 ☐
**Information support:** Sending informative messages
**Social support:** Online mood checkup
**Esteem support:** Sending automated inspirational messages
**Sense of belonging:** Chat groups
**Material aid:** medication reminder

Option 12 ☐
**Information support:** Sending instructional videos
**Social support:** Online mood checkup
**Esteem support:** Sending automated inspirational messages
**Sense of belonging:** Chat groups
**Material aid:** Send someone to help

***

Option 13 ☐
**Information support:** Sending informative messages
**Social support:** Sending automated caring messages
**Esteem support:** Offer mindfulness and positive affirmations
**Sense of belonging:** Chat groups
**Material aid:** medication reminder

Option 14 ☐
**Information support:** Sending instructional videos
**Social support:** Sending automated caring messages
**Esteem support:** Offer mindfulness and positive affirmations
**Sense of belonging:** Chat groups
**Material aid:** Send someone to help

***

Option 15 ☐
**Information support:** Sending informative messages
**Social support:** Online mood checkup
**Esteem support:** Offer mindfulness and positive affirmations
**Sense of belonging:** Chat groups
**Material aid:** Send someone to help

Option 16 ☐
**Information support:** Sending instructional videos
**Social support:** Online mood checkup
**Esteem support:** Offer mindfulness and positive affirmations
**Sense of belonging:** Chat groups
**Material aid:** medication reminder

Age: ……. (years)

Marital status: Married ☐ Single ☐ Divorced ☐

Family size: ……

Educational level: non-academic education☐ Academic Education☐ Post-graduate education☐

Occupation: Employed ☐ non-employed☐ retired ☐
